# Orthographic Reading Deficits in Dyslexic Japanese Children: Examining the Transposed-Letter Effect in the Color-Word Stroop Paradigm

**DOI:** 10.3389/fpsyg.2016.00767

**Published:** 2016-05-31

**Authors:** Shino Ogawa, Masahiro Shibasaki, Tomoko Isomura, Nobuo Masataka

**Affiliations:** ^1^Graduate School of Human and Environmental Studies, Kyoto UniversityKyoto, Japan; ^2^Faculty of Intercultural Studies, Nagoya Gakuin UniversityAichi, Japan; ^3^Graduate School of Frontier Biosciences, Osaka UniversityOsaka, Japan; ^4^Section of Cognition and Learning, Primate Research Institute, Kyoto UniversityAichi, Japan

**Keywords:** dyslexia, Japanese, orthographic reading, Stroop, transposed-letter effect

## Abstract

In orthographic reading, the transposed-letter effect (TLE) is the perception of a transposed-letter position word such as “cholocate” as the correct word “chocolate.” Although previous studies on dyslexic children using alphabetic languages have reported such orthographic reading deficits, the extent of orthographic reading impairment in dyslexic Japanese children has remained unknown. This study examined the TLE in dyslexic Japanese children using the color-word Stroop paradigm comprising congruent and incongruent Japanese hiragana words with correct and transposed-letter positions. We found that typically developed children exhibited Stroop effects in Japanese hiragana words with both correct and transposed-letter positions, thus indicating the presence of TLE. In contrast, dyslexic children indicated Stroop effects in correct letter positions in Japanese words but not in transposed, which indicated an absence of the TLE. These results suggest that dyslexic Japanese children, similar to dyslexic children using alphabetic languages, may also have a problem with orthographic reading.

## Introduction

Dyslexia is a developmental disorder characterized by reading difficulty in children and adults of normal intelligence who have the motivation to read accurately and fluently (Shaywitz and Shaywitz, 2005). The prevalence of dyslexia varies depending on the linguistic system. For instance, dyslexia is estimated in ~5–12% of participants who used English as a primary language (Katusic et al., 2001). Dyslexia has also been found in participants with non-alphabetic languages, such as Japanese, but at much lower percentages (Uno et al., 2009). This suggests that differences in the architecture of English and Japanese may be associated with a propensity to dyslexia at least partly; therefore, research that compares dyslexia in different language systems may provide important insights into its mechanism.

The underlying mechanisms of dyslexia have thus far remained largely unclear (Gabrieli, 2009; Dehaene et al., 2010). According to Coltheart's dual-route model (Coltheart et al., 2001), written words are processed in either lexical (orthographic) or sub-lexical (phonological) reading routes. For dyslexic users of alphabetic languages, both these routes are believed to be impaired (Gabrieli, 2009; Peterson and Pennington, 2012). In phonological reading, dyslexia can cause deficits in both the segmentation of a speech stream into phonological units and the association of each unit with its corresponding letter (Shaywitz and Shaywitz, 2005). Japanese writing has three different character systems: hiragana, katakana, and kanji. Dyslexia has been estimated to occur in ~0.2, 1.4, and 6.9%, for each system respectively (Uno et al., 2009). Previous research has reported that Japanese school-age children with kana (hiragana and katakana) dyslexia have difficulty associating each phonological unit with its corresponding letter but have no difficulty segmenting the speech stream into phonological units (Ogawa et al., 2014). This suggests that different mechanisms may be involved in English and Japanese dyslexia. These could be associated with some of the characteristics of kana, such as its psycholinguistic grain size and orthography-to-phonology translation relationships, that are quite different from alphabets (Wydell and Butterworth, 1999).

Previous studies involving alphabetic languages have reported orthographic process deficits in dyslexics (O'Brien et al., 2011; Kezilas et al., 2014; Ziegler et al., 2014). Some Japanese dyslexics have also exhibited difficulty in reading texts after acquiring a reading knowledge of kana characters (Yoshida and Tsuzuki, 2015). As most kana characters express one sound (Wydell and Butterworth, 1999), any difficulties faced after acquiring a reading knowledge of kana characters cannot be explained by phonological reading deficits. One possibility is that dyslexic children may also face difficulties with the orthographic process; that is, recognizing a word as a whole, along the lexical reading route. However, whether orthographic reading is also impaired in Japanese dyslexics remains unclear. In orthographic reading, letter positions within a word can be loosely perceived (Carreiras et al., 2015). For instance, a transposed-letter nonword such as “cholocate” is frequently misperceived as the word “chocolate.” This transposed-letter effect (TLE) has been reported in various European and non-European alphabetic languages, such as English (Perea and Lupker, 2003; Johnson et al., 2007), French (Schoonbaert and Grainger, 2004), Spanish (Perea and Carreiras, 2006a,b), and Basque (Duñabeitia et al., 2007). The TLE can be assessed using the color-word Stroop paradigm (Arsalidou et al., 2013). Arsalidou et al. (2013) revealed that the Stroop effect, that is, the interference observed when a color word and the actual printed color of the word are incongruent (e.g., the word “red” printed in blue), was observed in correct words (e.g., purple) as well as in transposed-letter nonwords (e.g., prulpe) in English (Arsalidou et al., 2013). Therefore, orthographic reading deficits can be evaluated by the presence or absence of the TLE in the color-word Stroop paradigm.

In this study, we examined orthographic reading deficits in dyslexic Japanese children with TLE using the color-word Stroop paradigm. To confirm the suitability of this experimental procedure (Experiment 1), we first examined whether TLE was observed in normal Japanese adults reading Japanese kana words using the color-word Stroop test. Thereafter, we examined orthographic reading impairments in dyslexic Japanese children (Experiment 2). If these children suffered from orthographic reading deficits, they were not expected to display or display a small TLE compared with normal Japanese children.

## Experiment 1

### Materials and methods

#### Ethics note

This study was conducted in accordance with the principles expressed in the Declaration of Helsinki and the Ethical Guidelines for Medical and Health Research Involving Human Subjects by the Japanese Ministry of Health, Labour, and Welfare. All experimental protocols were approved by the Institutional Ethics Committee of the Primate Research Institute, Kyoto University (permission number, H2012-09).

#### Participants

Participants included 22 Japanese adults (11 males and 11 females; mean age = 26.37; *SD* = 3.55) with no psychiatric or neurological conditions. All had normal or corrected-to-normal visual acuity and adequate color vision. All participants provided informed written consent to participate in this study.

#### Color-word stroop test

**Figure 1:**
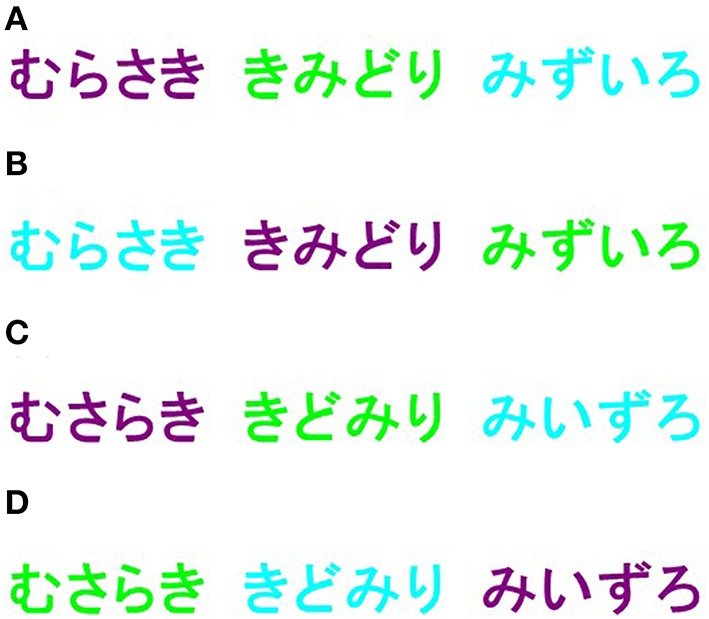
**Four conditions used in the color-word Stroop test**. In the color-word Stroop test, three color (purple, lime, aqua) words were employed. The following four conditions were utilized: **(A)** correct words written in Japanese hiragana with congruent color (e.g., “purple” written in purple ink); **(B)** correct words with incongruent color (e.g., “purple” written in lime ink); **(C)** transposed-letter nonwords with congruent color (e.g., “prulpe” written in purple ink); and **(D)** transposed-letter nonwords with incongruent color (e.g., “prulpe” written in lime ink).

In the color-word Stroop test (Stroop, 1935), participants are asked to name the ink color in which a congruent color word is written (i.e., the word “red” written in red ink: congruent condition) or the ink color of an incongruent color word (i.e., the word “blue” written in red ink: incongruent condition). Stroop effects are determined by comparing the reaction time (RT) in the incongruent condition with the RT in the congruent condition. Three colors were used for the Japanese characters in this test. Given that creating transposed-letter nonwords requires a relatively longer word length (four Japanese characters), purple [RGB (128, 0, 128)], lime [RGB (0, 255, 0)], and aqua [RGB (0, 255, 255)] were selected, which are commonly used colors that all participants were familiar with (Figure 1).

All participants could read and name the colors accurately. The test was conducted as a computerized manual response paradigm controlled by a custom-written software, Visual Basic 6.0 (Microsoft Corporation, Redmond, Washington, USA), and was run on a personal computer so that each participant pressed a button in response when they saw the stimulus on the screen. In this study, the RT was measured using a manual response paradigm.

To familiarize the participants with the test, one 20-trial training session was conducted before the actual test session. Stimuli created with MS P Gothic font, size 72, bold style, were presented without time limits in the center of a color monitor's visual field ~30 cm from the participants. During inter-trial intervals, a fixation mark (a white plus sign) was shown in the center of the visual field. Inter-trial intervals varied pseudo-randomly between 1500 and 2000 ms (increments of 100 ms). Responses were provided by pressing pre-defined buttons on the 10-digit keyboard without feedback. Colored stickers were placed on the relevant keys to reduce memory demands. Each participant's matching of colored stickers and key positions were randomly assigned. Participants were instructed to respond to the ink color of the stimuli as quickly and as accurately as possible. Stimuli included the following four conditions: (a) correct words written in Japanese hiragana with congruent color (e.g., “purple” written in purple ink); (b) correct words with incongruent color (e.g., “purple” written in lime ink); (c) transposed-letter nonwords with congruent color (e.g., “prulpe” written in purple ink); and (d) transposed-letter nonwords with incongruent color (e.g., “prulpe” written in lime ink) (Figure 1). In the transposed-letter nonwords, the word's first and last letters were kept in place while the middle letters were transposed. The four different stimuli conditions were pseudo-randomly presented with equal frequency (18 trials for each condition), thus resulting in 72 trials per session. Both accuracy and RTs were recorded.

#### Data analysis

Mean RTs were calculated for the trials with correct responses. RTs in trials greater and smaller than 2 SD from each participant's mean were eliminated from subsequent analysis as outliers (5.05%, *SD* = 1.39).

The Stroop effect, which was used as a dependent variable in the statistical analyses, was calculated using the following formula: [Incongruent RT/Congruent RT] (cf. [(Incongruent RT − Congruent RT)/Incongruent RT × 100] (Mayas et al., 2012), [(Incongruent RT − Congruent RT)/Congruent RT × 100] (Naccache et al., 2005).

The manipulation of the letter positions that affected the Stroop effect were examined using an analysis of covariance (ANCOVA) on the Stroop effects for the correct and transposed-letter words, with the WORD CONDITION (correct word vs. transposed-letter nonword) as the fixed within-subject factor and controlled for gender and individual error rate. Furthermore, to examine whether the Stroop effect itself existed, we ran a one-sample *t*-test on the Stroop effect for the correct or transposed-letter words. Statistical analyses were conducted using the freeware “R 3.2.3” (R Development Core Team) as “SPSS Statistics 22” (IBM Japan, Ltd).

### Results

**Figure 2:**
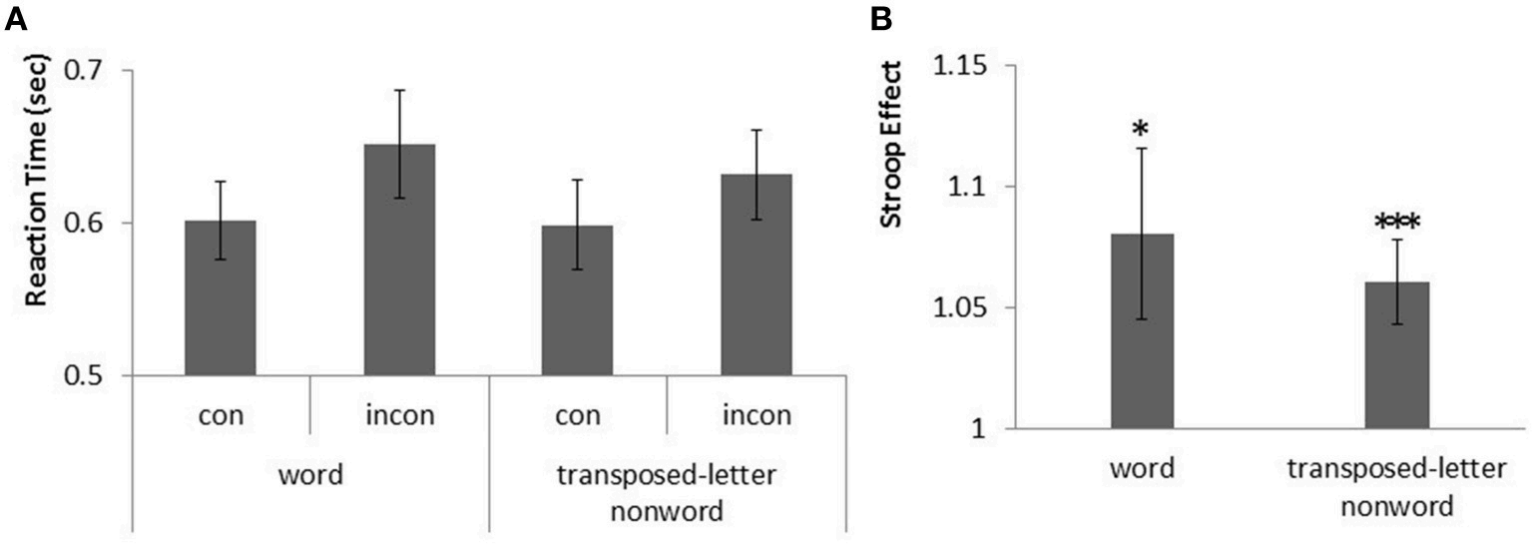
**Stroop effects for correct hiragana words and transposed-letter hiragana nonwords in normal adults. (A)** Graph showing RTs for the congruent (con) and incongruent (incon) conditions of the correct words and transposed-letter hiragana nonwords. The error bars indicate standard errors. **(B)** Similar graph showing Stroop effects for the correct words and the transposed-letter hiragana nonwords. The asterisks represent a significant difference (^*^p < 0.05, ^***^p < 0.001).

The average error rate was 3.47% (*SD* = 4.28). The mean RTs with correct and transposed-letter positions are shown in Figure 2A, and the Stroop effects for correct words and transposed-letter nonwords are shown in Figure 2B.

The ANCOVA results were as follows: as a parameter, gender showed no significance in the regression for both correct word condition [β = 0.08, *p* = 0.24] and transposed-letter nonword condition [β = 0.04, *p* = 0.21]; the error rate also showed no significance in the regression for both correct word condition [β = 1.34, *p* = 0.11] and transposed-letter nonword condition [β = 0.36, *p* = 0.40].

As no significance was observed in the regression for either gender error rate, we ran a paired *t*-test on the Stroop effects of the correct and transposed-letter words with WORD CONDITION (correct word vs. transposed-letter nonword). The Stroop effects showed no difference between the word and transposed-letter nonword [*t*_(21)_ = 0.61, *p* = 0.54].

The one-sample *t*-test revealed statistically significant Stroop effects in both the correct words [*t*_(21)_ = 2.27, *p* = 0.01, Cohen's *d* = 0.34] and the transposed-letter words [*t*_(21)_ = 3.43, *p* = 0.001, Cohen's *d* = 0.23].

### Discussion

In this experiment, Stroop effects were observed for both correct words and transposed-letter nonwords, and neither error rate nor gender confounded the Stroop effects in adults. Therefore, these results suggested that normal Japanese adults utilize orthographic reading when recognizing transposed-letter nonwords in the color-word Stroop paradigm.

## Experiment 2

### Materials and methods

#### Participants

Participants were 20 typically developing (TD) children (11 male and 9 female) (mean age = 11.27; SD = 1.01; range = 10.08–13.00) without psychiatric or neurological conditions, and 11 dyslexic children (7 male and 4 female) (mean age = 11.65; SD = 0.35; range = 10.91–13.00). Table 1 summarizes the information on the dyslexic children, indicating their gender, full-scale IQ, and age. All participants had been diagnosed by a child psychiatrist at a general hospital or a child consultation center, and all demonstrated difficulty in reading Japanese compared to their peers. They had been receiving special training because of their dyslexia for 1 to 4 years, and they could therefore read hiragana words well when there were limited words shown; however, they could not correctly read many words in longer texts. The Intelligence Quotient (IQ) was measured using the Japanese version of the Wechsler Intelligence Scale for Children (either WISC-III or WISC-IV). Age [*t*_(29)_ = −1.14, *p* = 0.26] and Full-scale IQ [*t*_(29)_ = 1.94, *p* = 0.06] and was the same for both the TD and dyslexic participants. All participants had normal or corrected-to-normal visual acuity and adequate color vision. The participants' parents provided informed written consent for their children's participation in this study.

**Table 1:** **Information on the dyslexic children in the study**.

**ID**	**Gender**	**FIQ**	**Age**
1	Female	101	13 y 0 m
2	Male	100	12 y 0 m
3	Male	104	12 y 0 m
4	Male	79	12 y 0 m
5	Male	103	11 y 11 m
6	Female	90	11 y 6 m
7	Male	97	11 y 4 m
8	Female	101	11 y 3 m
9	Female	112	11 y 2 m
10	Male	88	11 y 2 m
11	Male	93	10 y 11 m

#### Color-word stroop test

The same color-word Stroop test as described in Experiment 1 was used. All participants could accurately read and name the colors. When the children demanded breaks during the test, short breaks of no longer than 10 min were given.

#### Data analysis

Mean RTs were calculated for trials with correct responses. RTs >4 s and RTs in trials greater and smaller than 2 SD from each participant's mean were eliminated from subsequent analysis as outliers (TD children: 4.72%, *SD* = 1.71; dyslexic children: 7.07%, *SD* = 2.73).

The Stroop effects as a dependent variable were calculated using the following formula: [Incongruent RT/Congruent RT] (cf. [(Incongruent RT − Congruent RT)/Incongruent RT × 100] (Mayas et al., 2012), [(Incongruent RT − Congruent RT)/Congruent RT × 100] (Naccache et al., 2005).

To examine the Stroop effect in each word condition for the TD/dyslexic participants, we ran an ANCOVA on the Stroop effects of the correct and transposed-letter words with WORD CONDITION (correct word vs. transposed-letter nonword) as the fixed within-subject factor and GROUP CONDITION (TD vs. dyslexia) as the fixed between-subject factor, with gender, error rate, FIQ, and age as covariates. To examine whether the Stroop effect exised, we ran a one-sample *t*-test on the Stroop effect of the correct or transposed-letter words. Statistical analyses were conducted using the freeware “R 3.2.3” (R Development Core Team) as “SPSS Statistics 22” (IBM Japan, Ltd).

### Results

The average error rates were 4.44% (*SD* = 3.32) in the TD children and 4.29% (*SD* = 5.57) in the dyslexic children; therefore, no significant difference was observed between the groups [*t*_(29)_ = −0.09, *p* = 0.92].

**Figure 3:**
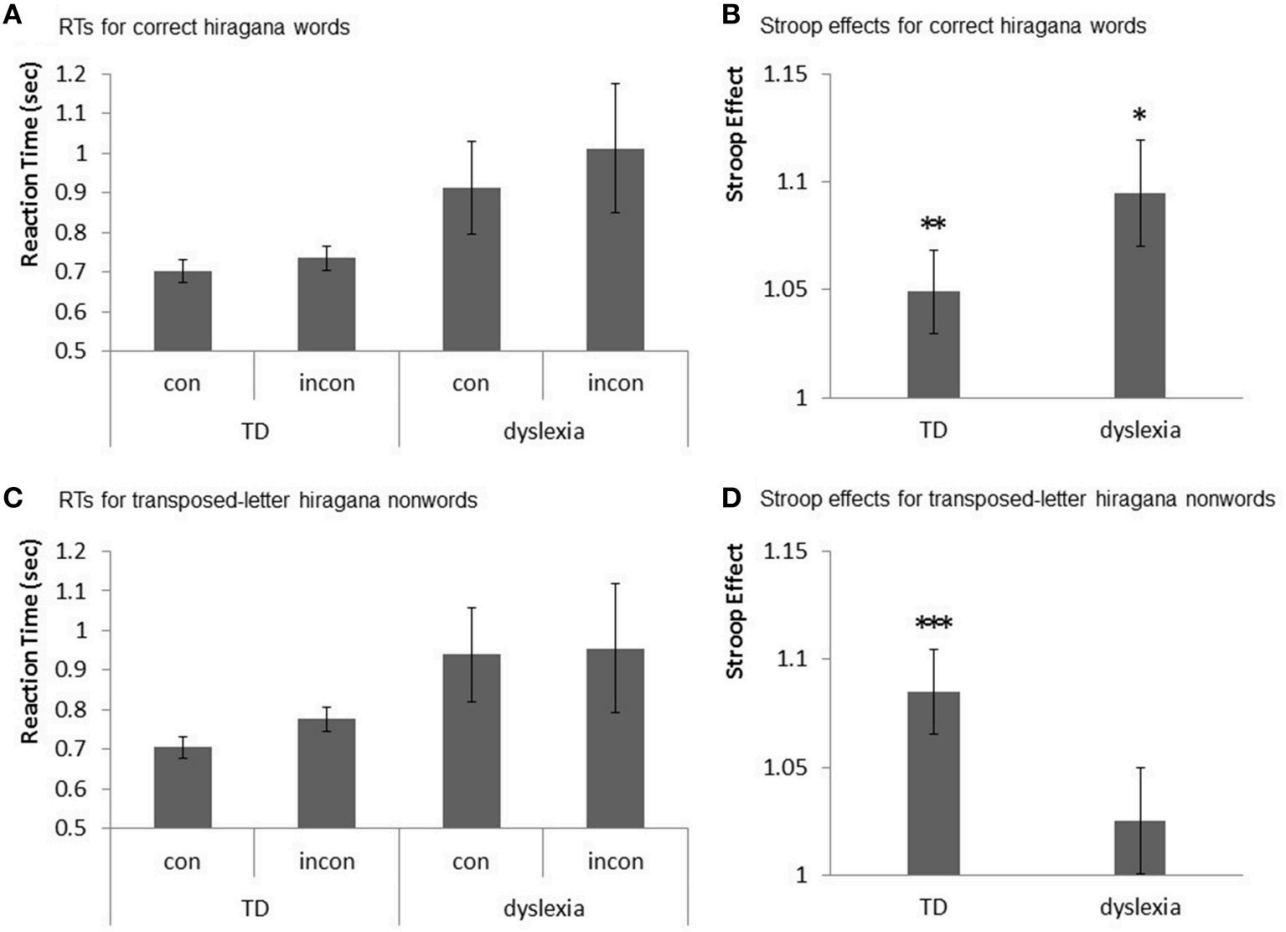
**Stroop effects for correct hiragana words and transposed-letter hiragana nonwords in TD and dyslexic children. (A)** Graph showing the RTs for the congruent (con) and incongruent (incon) conditions of the correct hiragana words with TD and dyslexic children. **(B)** Graph showing the Stroop effects for the correct hiragana words with TD and dyslexic children. The asterisks represent a significant difference (^*^p < 0.05, ^**^p < 0.01). **(C)** Graph showing the RTs for the congruent (con) and incongruent (incon) conditions for the transposed-letter hiragana nonwords with TD and dyslexic children. **(D)** Graph showing the Stroop effects for the transposed-letter hiragana nonwords in TD children, but not in dyslexic children. The asterisk represents a significant difference (^***^p < 0.001).

Mean correct word RTs for the TD and dyslexic children are shown in Figure 3A, and the Stroop effects in the TD and dyslexic children are shown in Figure 3B. In addition, Mean transposed-letter nonwords RTs for the TD and dyslexic children are shown in Figure 3C, and the Stroop effects in TD and dyslexic children are shown in Figure 3D.

The ANCOVA results were as follows. There were regression parallels between GROUP CONDITION and gender [*F*_(1, 21)_ = 2.50, *p* = 0.12], GROUP CONDITION and error rate [*F*_(1, 21)_ = 0.40, *p* = 0.53], GROUP CONDITION, and FIQ [*F*_(1.21)_ = 0.29, *p* = 0.59], and GROUP CONDITION and age [*F*_(1, 21)_ = 0.28, *p* = 0.59]. Gender showed no significance in the regression for the transposed-letter nonword condition [β = −0.015, *p* = 0.70]; however, it was significant in the regression for the correct word condition [β = −0.068, *p* = 0.09]. Error rate, similarly, showed no significance in the regression for the transposed-letter nonword condition [β = 0.134, *p* = 0.24] but was significant in the regression for the correct word condition [β = −0.794, *p* = 0.07]. The FIQ showed no significance in the regression for both the correct word condition [β = 0.002, *p* = 0.27] and the transposed-letter nonword condition [β = 0.000, *p* = 0.83]. Age showed no significance in the regression for the correct word condition [β = −0.003, *p* = 0.87] but was significant in the regression for the transposed-letter nonword condition [β = −0.044, *p* = 0.04].

As no significance was observed in the FIQ in the regression, the interaction between WORD CONDITION and gender, error rate, or age, we ran an ANOVA on the Stroop effects of the correct and transposed-letter words with WORD CONDITION (correct word vs. transposed-letter nonword) as the within-subject factor and GROUP CONDITION (TD vs. dyslexia) as the between-subject factor.

Table 2 summarizes the variance sources. No main effect was observed for GROUP CONDITION [*F*_(1, 29)_ = 0.36, *p* = 0.55, $\eta_{G}^{2}$ = 0.004] or WORD CONDITION [*F*_(1, 29)_ = 0.06, *p* = 0.80, $\eta_{G}^{2}$ = 0.001]. A significant trend was observed in the interaction between GROUP CONDITION and WORD CONDITION [*F*_(1, 29)_ = 3.72, *p* = 0.06, $\eta_{G}^{2}$ = 0.039].

**Table 2:** **Summary of variance sources tested using a TD and dyslexic children ANOVA**.

**Source**	***df***	***F***	***p***	***$\eta_{G}^{2}$***
GROUP CONDITION	1	0.36	0.55	0.004
WORD CONDITION	1	0.06	0.80	0.001
GROUP CONDITION × WORD CONDITION	1	3.72	0.06	0.039

The symbol $\eta_{G}^{\text{2}}$ represents generalized eta squared statistics.

The simple main effect of the WORD CONDITION was not significant for either the TD children [*F*_(1, 29)_ = 1.97, *p* = 0.17] or the dyslexic children [*F*_(1, 29)_ = 1.84, *p* = 0.18]. However, the simple main effect of the GROUP CONDITION was marginally significant for the transposed-letter nonword condition [*F*_(1, 29)_ = 3.43, *p* = 0.07] but not for the correct word condition [*F*_(1, 29)_ = 0.77, *p* = 0.38].

We used a one-sample *t*-test to examine whether the Stroop effect existed for each condition. For the correct word, the one-sample *t*-test revealed statistically significant effects in both the TD children [*t*_(19)_ = 2.50, *p* = 0.01, Cohen's *d* = 0.24] and the dyslexic children [*t*_(10)_ = 2.02, *p* = 0.03, Cohen's *d* = 0.20]. For the transposed-letter nonword, the one-sample *t*-test revealed statistically significant effects in the TD children [*t*_(19)_ = 3.87, *p* = 0.0005, Cohen's *d* = 0.46) but not in the dyslexic children (*t*_(10)_ = 1.06, *p* = 0.15, Cohen's *d* = 0.03).

### Discussion

In Experiment 2, TD and dyslexic children showed different Stroop effect tendencies for the correct word and transposed-letter nonword tasks. Dyslexic children showed a Stroop effect in the correct word condition but not in the transposed-letter nonword condition; however, the TD children indicated clear Stroop effects for both the word and transposed-letter nonword conditions. This result suggests that the dyslexic children who participated in this study can use phonological reading but face problems with orthographic reading. This does not contradict the fact that the dyslexic children participating in this study could read hiragana words well when there were limited words shown but could not read several words in longer texts correctly.

If the hypothesis that dyslexic children were not expected to display or display a small TLE compared with normal Japanese children is true, the interaction between GROUP CONDITION and WORD CONDITION should be significant. In this experiment, the interaction was marginally significant but failed to achieve statistical significance. This may be attributed to the small sample size or large variance of dyslexic children. Because of many individual differences in dyslexic children, larger sample sizes will be required in future studies.

## General discussion

In this study, we found that both normal adults and TD children exhibited Stroop effects in Japanese hiragana words with transposed-letter positions, indicating the presence of TLE in orthographic reading. In contrast, Stroop effects for words with transposed-letter positions were not observed in dyslexic children, which was consistent with previous research on dyslexic children in other languages (O'Brien et al., 2011; Kezilas et al., 2014; Ziegler et al., 2014) and indicated that dyslexic Japanese children may also face difficulties in orthographic reading.

The current study demonstrated the TLE in Japanese hiragana using the Stroop paradigm, as has been previously shown for English (Arsalidou et al., 2013). Although, the unit size (syllable) of hiragana is greater than the unit size (phoneme) for the Roman alphabet, the letter position of a hiragana word may affect its orthographic readability similarly to that of Western languages that use the Roman alphabet. Consistent with our findings, two previous studies have examined TLE in Japanese kana using different experimental paradigms (Perea and Perez, 2009; Perea et al., 2011). Using a masked priming lexical decision paradigm, Perea and Perez (2009) reported that the lexical decision time for the word “a.me.ri.ka [
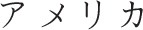
]” was faster when the prime was a transposed-letter nonword “a.ri.me.ka [
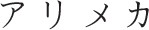
]” than the control nonword “a.ka.ho.ka [
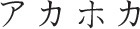
].” Perea et al. (2011) also found that while using the silent reading paradigm, fixation time on the target word was shorter when the parafoveal preview was the transposed-letter nonword (a.ri.me.ka [
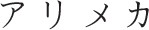
]–a.me.ri.ka [
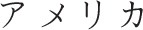
]) than the control nonword (a.ka.ho.ka [
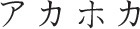
] –a.me.ri.ka [
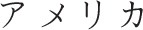
]). A major limitation of these studies was that the experimental procedures often used unfamiliar words, especially for young children, it was therefore difficult to distinguish whether the low task performance was due to orthographic reading deficits or whether the words used in the task were unfamiliar to the participants. The color-word Stroop paradigm overcomes this problem as the words used in this paradigm can be fixed to only include familiar words.

People showing TLE can recognize letters with a high abstraction level. For people who have difficulty with the orthographic process, it may be difficult to recognize the same character or letters in different fonts or handwritten characters. This could be supporting evidence for why dyslexics have difficulty reading even after they have acquired the characters or words.

This is the first report suggesting that orthographic reading is possibly impaired in Japanese dyslexics. From this and our previous study (Ogawa et al., 2014), Japanese dyslexics have been found to struggle with both phonological and orthographic reading, as in dyslexics from alphabetic language backgrounds (Gabrieli, 2009; Peterson and Pennington, 2012), although the percentage of dyslexics in Japan is much lower than in countries with speakers of alphabetic languages (Uno et al., 2009).

In particular, these results are very important for understanding and supporting dyslexic Japanese children who have difficulty reading texts even after having learnt how to read the kana characters, which cannot be explained by phonological reading deficits. This study suggests that these children may have difficulty with the orthographic process and may need special support specific to whole word recognition. This study included a small sample of dyslexic children. In the future, to ensure generalizability, larger sample sizes, and additional tests are required.

In conclusion, our study suggested that dyslexic Japanese children have difficulty in orthographic reading and that the Stroop paradigm was a useful tool in assessing the orthographic process for Japanese dyslexics.

## Author contributions

SO designed the study, and prepared the manuscript. MS programmed the computer-based task. SO, MS, and TI conducted the study. SO, MS, TI, and NM analyzed the data. SO, MS, TI, and NM prepared the manuscript.

### Conflict of interest statement

The authors declare that the research was conducted in the absence of any commercial or financial relationships that could be construed as a potential conflict of interest.
